# Supplementary material for: Visualization of cytosolic ribosomes on the surface of mitochondria by electron cryo‐tomography
Source: EMBO Rep. 2017 Aug 21;18(10):1786–800. doi: 10.15252/embr.201744261 (PMC5623831; doi:10.15252/embr.201744261)
Supplement: Supplementary file 1 — Expanded View Figures PDF [file EMBR-18-0-s001.pdf]

Expanded View Figures

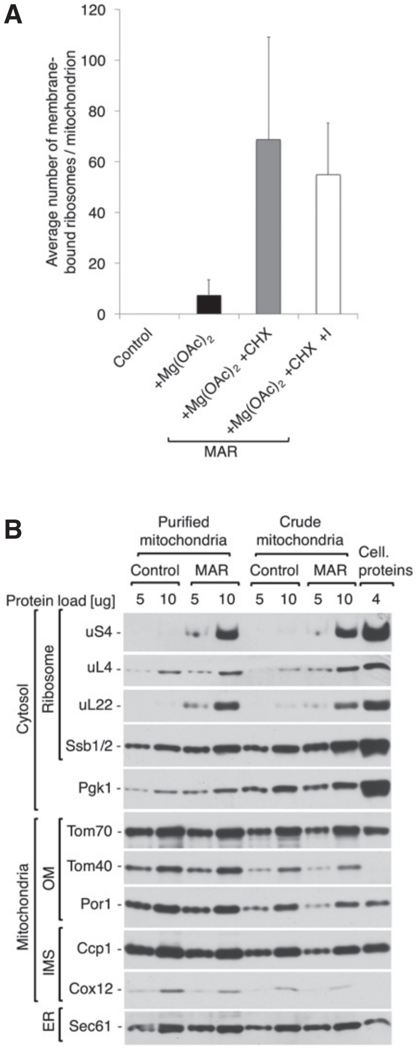

Figure EV1. Step gradient purification reduces cytosolic and ER contamination but does not alter ribosome content in MAR samples.

A Average number of ribosomes bound to mitochondria for control (−Mg $(OAc)_2$ −CHX), +Mg$(OAc)_2$ only, and two MAR (+Mg$(OAc)_2$ +CHX) samples, from a crude isolation and iodixanol purification (+I). Data are plotted as the mean number of ribosomes per mitochondrion ± SEM. $n = 28$ mitochondria, combined from > 10 independent sample preparations.

B The steady-state protein levels of crude or step gradient-purified mitochondria and MAR isolated from cells. Step gradient-purified samples are enriched with mitochondrial marker proteins in contrast to cytosolic protein Pgk1. Cellular protein extract (4 μg) was loaded as a reference. Samples were analyzed by SDS–PAGE followed by immunodecoration with specific antisera. OM, outer membrane; IMS, intermembrane space; ER, endoplasmic reticulum.

© 2017 The Authors

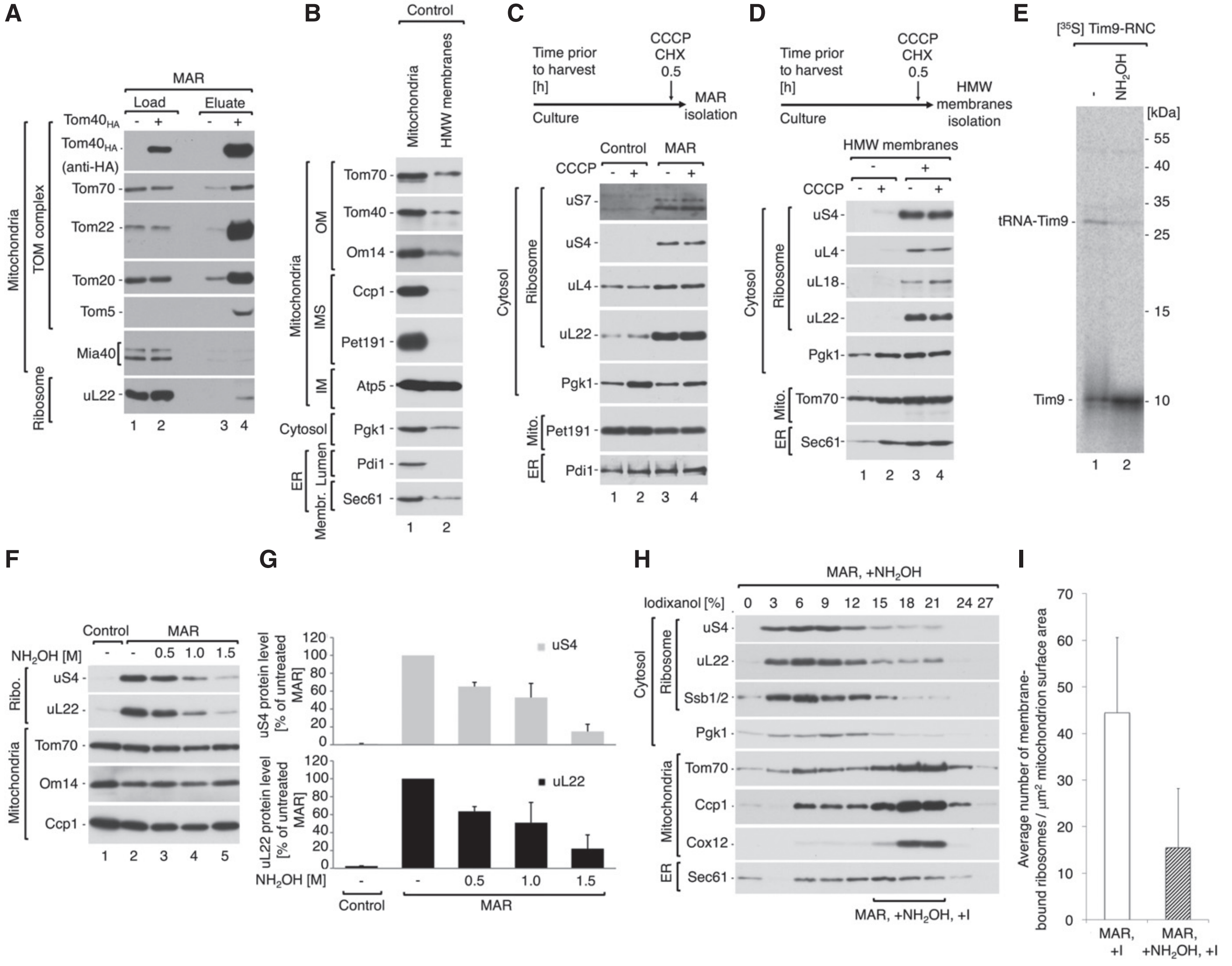

Figure EV2. Cytosolic ribosomes are stabilized on mitochondria via the nascent chain.

A Immuno-affinity purification of $Tom40_{HA}$ from digitonin-solubilized MAR. Protein uL22 co-purifies with the TOM complex. Load 4%; eluate 100%.

B The steady-state protein level of crude mitochondria and HMW membranes indicates the presence of mitochondrial and ER membranes in both samples. Equal mass of protein (10 µg) was compared. Due to whole cell rupture, the HMW membrane fraction contains membrane proteins, but not soluble proteins, from the IMS and ER lumen. OM, outer membrane; IMS, intermembrane space; IM, inner membrane.

C, D The steady-state protein levels of crude mitochondria and MAR (C) or corresponding HMW membranes (D) isolated from yeast cells treated with 10 µM CCCP and 50 µg/ml CHX 30 min prior to harvest. Ribosomes are stabilized irrespective of the inner mitochondrial membrane potential.

E [^{35}S]-labeled Tim9-RNC was treated with 1.5 M hydroxylamine and analyzed by SDS–PAGE followed by autoradiography. Hydroxylamine causes polypeptide nascent chain release from RNCs.

F Protein levels in MAR samples upon treatment with increasing concentration of hydroxylamine in SM buffer.

G Quantification of the ribosomal protein levels from samples shown in (F). The protein levels of uS4 and uL22 in MAR were set to 100%. Data are presented as the mean ± SEM. n = 3 biological replicates.

H Fractionation of MAR samples after incubation with 1.5 M hydroxylamine (MAR, +NH_2OH) in a 0–27% iodixanol step gradient. Iodixanol gradient-purified MAR (MAR, +NH_2OH, +I) were isolated from 15 to 21% iodixanol layers. Co-sedimentation of ribosomal markers with cytosolic protein Pgk1 indicates efficient dissociation of ribosomes from the mitochondrial outer membrane.

I Average number of ribosomes bound to mitochondria for iodixanol-purified MAR (MAR, +I) and hydroxylamine-treated MAR (MAR, +NH_2OH, + I) purifications. Data are presented as the mean ± SEM. n = 21 mitochondria (824 MAR, +I ribosomes from five independent sample preparations) and 126 MAR, +NH_2OH, + I ribosomes from a single sample preparation).

Data information: In (A–D, F, H), samples were analyzed by SDS–PAGE and Western blotting using specific antisera. ER, endoplasmic reticulum.

 © 2017 The Authors

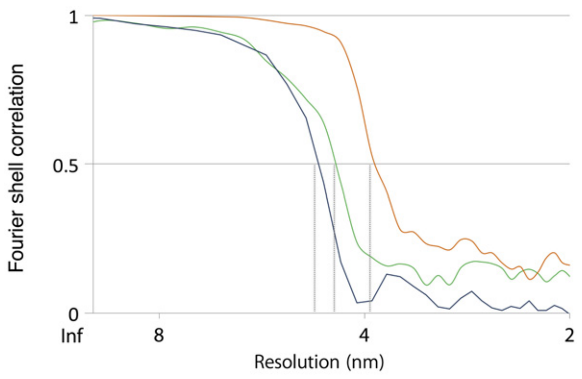

Figure EV3. Fourier shell correlation (FSC) curves for subtomogram averages.
Resolution estimates were based on conventional FSC measurements and the 0.5 criterion in IMOD. Calculations for MAR-M (orange, 38 Å), MAR-P (blue, 46 Å), and ER-R (green, 44 Å) are shown.

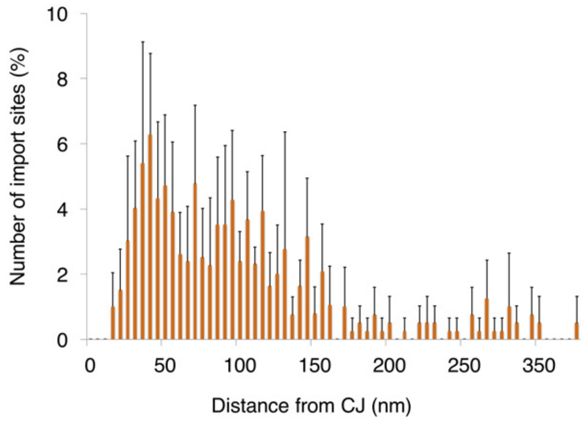

Figure EV4. Ribosomes cluster around the CJ.
Histogram showing closest-neighbor distribution distances between MAR-M and the nearest CJ, expressed in percent. Error bars indicate the standard deviation of the frequency distribution for each minimal distance. Data are obtained from two independent sample preparations.

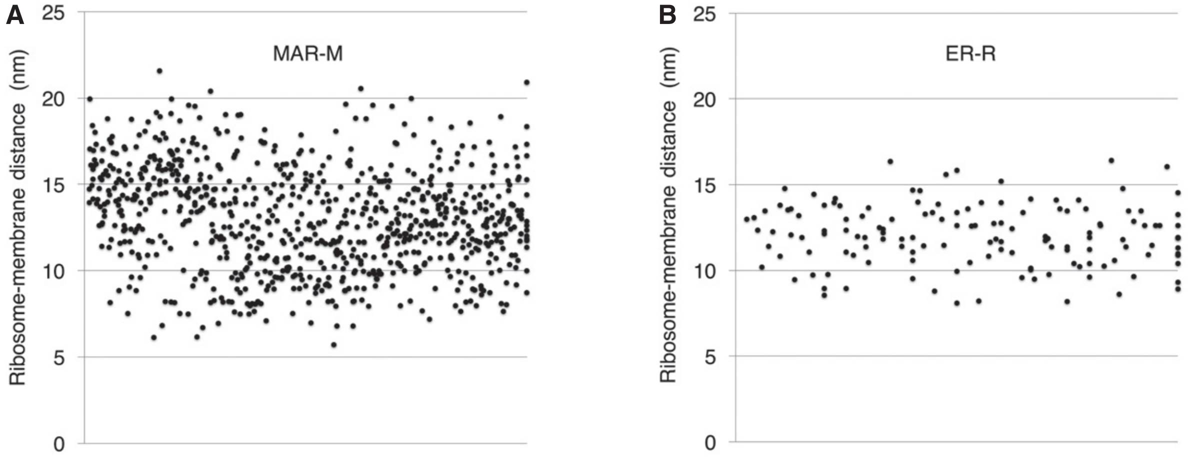

Figure EV5. Scatter plots showing the distance between ribosomes and the mitochondrial or ER membrane.
A, B All individual data points are plotted to demonstrate the degree of variation in the (A) MAR-M data series or (B) the ER-R group.

© 2017 The Authors
