# Supplementary material for: Visualization of cytosolic ribosomes on the surface of mitochondria by electron cryo‐tomography
Source: EMBO Rep. 2017 Aug 21;18(10):1786–800. doi: 10.15252/embr.201744261 (PMC5623831; doi:10.15252/embr.201744261)
Supplement: Supplementary file 2 — Movie EV1 [file EMBR-18-0-s002.zip › Movie_EV_1_legend.docx]

**Movie EV 1. Tomographic volume and segmentation of a mitochondrion with CHX-arrested ribosomes.**

The outer membrane (yellow) and a crista membrane (transparent) are shown relative to the position of MAR-M (60S subunit yellow, 40S subunit orange) and MAR-P (60S subunit light blue, 40S subunit dark blue). Note that not all crista membranes are shown in the segmentation.
