# Supplementary figures and images for: Visualization of cytosolic ribosomes on the surface of mitochondria by electron cryo‐tomography
Source: EMBO Rep. 2017 Aug 21;18(10):1786–800. doi: 10.15252/embr.201744261 (PMC5623831; doi:10.15252/embr.201744261)

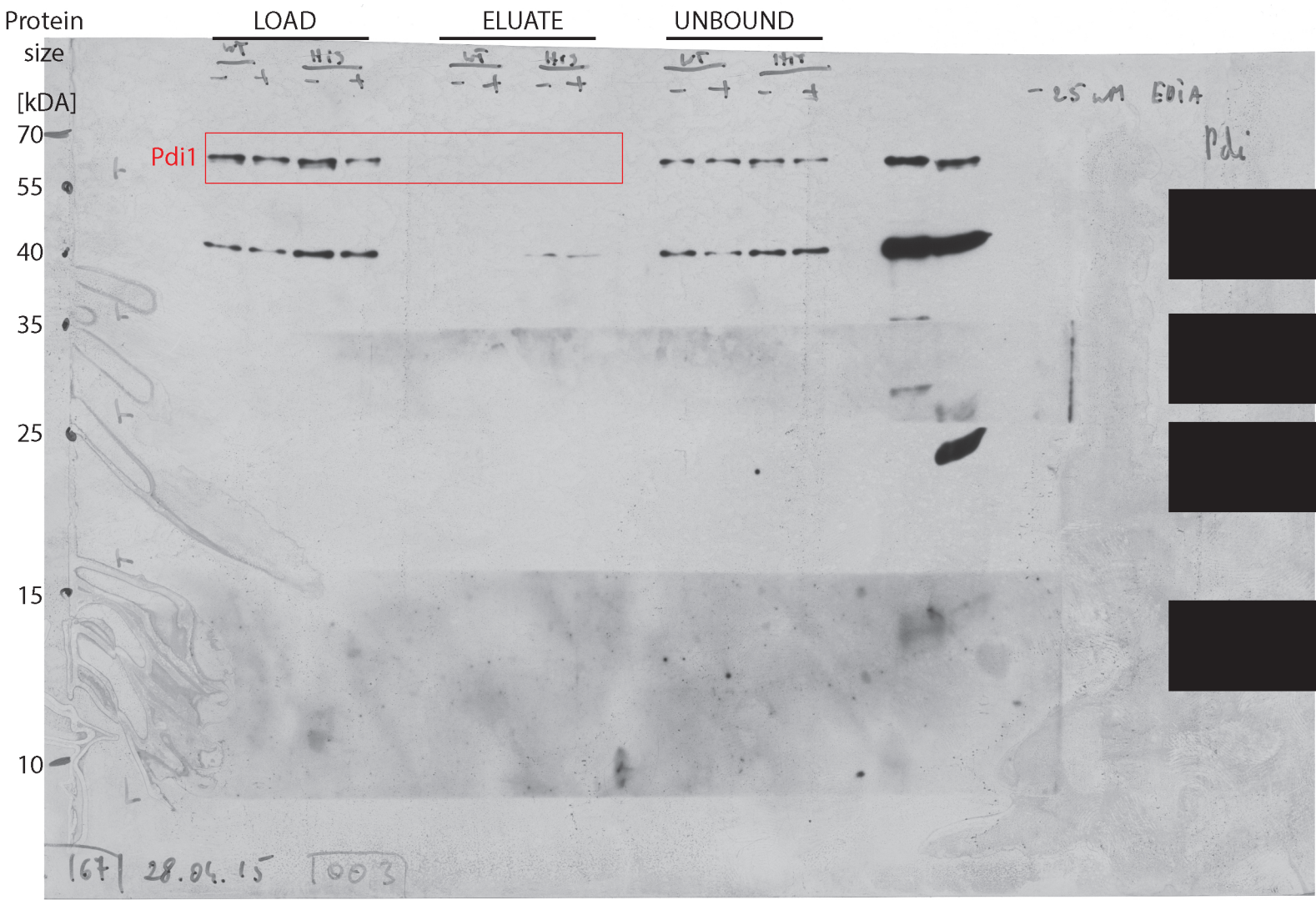

Supplement: Supplementary file 4 — Source Data for Figure 2A [file EMBR-18-0-s003.pdf]
